# The impact of HIV on the prevalence of asthma in Uganda: a general population survey

**DOI:** 10.1186/s12931-018-0898-5

**Published:** 2018-09-21

**Authors:** Bruce J. Kirenga, Levicatus Mugenyi, Corina de Jong, J. Lucian Davis, Winceslaus Katagira, Thys van der Molen, Moses R. Kamya, Marike Boezen

**Affiliations:** 1 Makerere University Lung Institute & Division of Pulmonary Medicine, Department of Medicine, Makerere University College of Health Sciences, Kampala, Uganda; 2 Makerere University Lung Institute, Makerere University College of Health Sciences, Kampala, Uganda; 3 GRIAC-Primary Care, Department of General Practice and Elderly Care, University of Groningen, University Medical Center Groningen (UMCG), Groningen, The Netherlands; 4 Groningen Research Institute for Asthma COPD (GRIAC), University of Groningen, University Medical Center Groningen (UMCG), Groningen, The Netherlands; 5 Department of Epidemiology of Microbial Diseases, Yale School of Public Health, and Pulmonary, Critical Care, and Sleep Medicine Section, Yale School of Medicine, New Haven, CT USA; 6 Makerere University Lung Institute, Makerere University College of Health Sciences, Kampala, Uganda; 7 Department of Medicine, Makerere University College of Health Sciences, Kampala, Uganda; 8 Department of Epidemiology, University of Groningen, Groningen, The Netherlands

**Keywords:** Asthma, HIV, Prevalence, Uganda

## Abstract

**Background:**

HIV and asthma are highly prevalent diseases in Africa but few studies have assessed the impact of HIV on asthma prevalence in high HIV burden settings. The objective of this analysis was to compare the prevalence of asthma among persons living with HIV (PLHIV) and those without HIV participating in the Uganda National Asthma Survey (UNAS).

**Methods:**

UNAS was a population-based survey of persons aged ≥12 years. Asthma was diagnosed based on either self-reported current wheeze concurrently or within the prior 12 months; physician diagnosis; or use of asthma medication. HIV was defined based on confidential self-report. We used Poisson regression with robust standard errors to estimate asthma prevalence and the prevalence ratio (PR) for HIV and asthma.

**Results:**

Of 3416 participants, 2067 (60.5%) knew their HIV status and 103 (5.0%) were PLHIV. Asthma prevalence was 15.5% among PLHIV and 9.1% among those without HIV, PR 1.72, (95%CI 1.07–2.75, *p* = 0.025). HIV modified the association of asthma with the following factors, PLHIV vs. not PLHIV: tobacco smoking (12% vs. 8%, *p* = < 0.001), biomass use (11% vs. 7%, *p* = < 0.001), allergy (17% vs. 11%, *p* = < 0.001), family history of asthma (17% vs. 11%, *p* = < 0.001), and prior TB treatment (15% vs. 10%, *p* = < 0.001).

**Conclusion:**

In Uganda the prevalence of asthma is higher in PLHIV than in those without HIV, and HIV interacts synergistically with other known asthma risk factors. Additional studies should explore the mechanisms underlying these associations. Clinicians should consider asthma as a possible diagnosis in PLHIV presenting with respiratory symptoms.

## Background

Human Immunodeficiency Virus (HIV) and asthma are both highly prevalent diseases globally [1, 2]. An estimated 334 million people have asthma and 36.7 million people have HIV [1, 2]. Both diseases disproportionately affect Africa and other low and middle income countries (LMICs) [2, 3]. The weighted mean prevalence of asthma in Africa is 7.0% in the rural areas (2.5–11.5) and 9.6% (3.9–15.2) in urban areas. The prevalence of asthma and HIV in Uganda is 10% and 6.2% respectively [4, 5].

Epidemiological studies have found increased prevalence of asthma among HIV infected persons [6–13]. However, the number of studies is small and most are either clinical or hospital based and most of them have been conducted in high income low HIV burden settings. Examples of available studies include a study that included 248 HIV infected and 236 HIV uninfected males. This study found that the prevalence of wheezing was 54.4%, vs. 21.2%, *p* < 0.001 [9]. In another study among 223 HIV patients in the USA, the prevalence of doctor diagnosed asthma was 20.6 compared to 8.2% in the general population [13]. In a study comparing 14,005 HIV infected with age matched HIV uninfected controls in Canada, the prevalence ratio for asthma was 1.31 (95% CI 1.20–1.43) [12].

Several mechanisms through which HIV increases the prevalence of asthma have been proposed [14–16]. Notable among these is the HIV associated persistent immune activation and inflammation [17]. It is also postulated that HIV proteins such as the nef protein or activation of memory CD4 T cells directly increase the risk of asthma [18]. Studies indicate that the higher the viral loads, the worse the lung function in HIV infected populations [14, 17]. HIV infected persons have been found to have higher levels of Immunoglobulin E (IgE) and this increases with increasing immunosuppression [15]. IgE is a well-known mediator of allergy and asthma. HIV could also drive asthma through its association with predominance of T-helper 2 (Th2) pathway. Priming of the Th2 pathway is known to increase the risk of asthma and other allergic diseases [19, 20]. In a cohort of 223 HIV infected persons, doctor diagnosed asthma appeared to be more common in participants with high sputum interleukin 4 (IL-4) (27% with asthma if high IL-4 vs.10.5% with asthma if low IL-4, *p* = 0.02) [13]. Antiretroviral therapy (ART) medications used to treat HIV have also be implicated in increasing the risk of asthma among HIV infected persons [21].

An interaction between HIV and asthma is important in high HIV burden settings because with the availability of highly active antiretroviral therapy and efficient health systems to deliver them, many HIV infected people are living longer into age groups where non-communicable diseases (NCDs) are common. In addition, prevention of HIV may lead to reduction in the burden of asthma. Data on the burden of asthma in HIV may also lead to assessment of asthma in HIV and routine HIV testing among persons with asthma. Drug-drug interactions in the management of patients with asthma-HIV comorbidity is also a key consideration.

In this study we analyzed data from the Uganda national asthma survey (UNAS) to determine if the prevalence of asthma was higher among persons with self-reported HIV infection. We also aimed to determine if HIV interacts with other known asthma risk factors namely tobacco smoking, biomass use, TB, family history of asthma and allergy.

## Methods

### Design and study participants

UNAS was a cross sectional general population-based survey conducted in Uganda in 2016. Participants were selected in clusters (villages) selected by probability proportionate to size by the Uganda Bureau of Statistics using the Uganda National population and housing census of 2014. Households (HHs) within clusters were selected by simple random sampling from a HH list generated by village leaders. All persons aged ≥12 years who were members of selected HH and provided written informed consent (and assent in case of minors) were surveyed. Exclusion criteria were: residing in congregate settings (schools, prisons, homes) and temporary residents (less than 2 weeks in household of selected villages).

### Asthma diagnosis

Sampled participants were interviewed by trained research assistants using a standardized questionnaire developed by adapting questions from internationally recognized questionnaires, including the World Health Organization (WHO) health survey [1, 22], the international study of asthma and allergies in children (ISAAC) [22] and the European community respiratory health survey (ECRHS) surveys [23]. Asthma was defined as self-report of physician diagnosis, prescribed use of breathing medications for asthma or report of a wheeze in the last 12 months.

### HIV diagnosis

HIV status in this survey was established by self-report. All participants were asked about their HIV status in private interviews. Responses were either HIV positive, HIV negative or unknown HIV status. HIV positive participants were classified as persons living with HIV (PLHIV).

### Statistical analysis

Participants with unknown HIV status were excluded from this analysis. Participants’ demographic and social characteristics and known asthma risk factors (tobacco smoking, allergy, family history of asthma, biomass use, and history of TB treatment) were summarized as proportions and compared between PLHIV and those who were not using Chi-square and Fisher’s exact test statistics as appropriate.

We used Poisson regression with robust standard errors to estimate asthma prevalence and prevalence ratios (PR) between PLHIV and those who were not [24]. The Poisson model, an alternative for the log-binomial, was used due to convergence problems with the latter approach. The prevalence of asthma and the corresponding PRs among patients with key asthma risk factors namely tobacco smoking, biomass smoke exposure, family history of asthma, tuberculosis (TB) and allergy were also calculated. The PR ratio of asthma between PLHIV and those who were not living with HIV was then finally adjusted for these risk factors. Exposure to biomass smoke was defined as cooking in the living space (same room where participants slept), tobacco smoking was by self-report of being a smoker while allergy was considered to be present if a participant reported suffering in the past 12 months from any of the following: suffering in the past 12 months from any of watery itchy eyes, recurrent skin rash, or having sneezing, nasal congestion or rhinorrhea in the absence of an upper respiratory tract infection). To assess for the interaction between HIV and known asthma risk factors (tobacco smoking, biomass use, history of TB treatment, allergy, family history), we calculated age-dependent asthma prevalence comparing PLHIV and those who were not while keeping all other factors at zero and then by each factor. A graphical aid was used to visualize the age-dependent asthma prevalence by these factors.

## Results

### Characteristics of study participants

Of 3416 UNAS participants, 2067 (60.5%) knew their HIV status and 103 reported to be living with HIV (5.0% of the group that knew their HIV status). The characteristics of these participants by HIV status are shown in Table 1. The proportions of participants with respiratory symptoms of cough, sputum production and wheezing did not differ by HIV status. However, the proportions with the symptom breathlessness differed significantly by HIV status, positive vs. negative (17.5% vs. 9.0%, *p* = 0.004). Tobacco smoking was higher among PLHIV (12.6% vs. 7.9%, *p* = 0.086) as well as having history of TB treatment (9.7% vs.1.5%, *p* < 0.001).

**Table 1:** Participant characteristics Please see my feedback in the previous file

	PLHIV (*N* = 103)	Not PLHIV (*N* = 1964)	*P*-value
Characteristic			
Male gender, *n*(%)	31 (30.1)	763 (38.8)	0.076
Age groups *n*(%)			< 0.001
< 15	1 (1.0)	91 (4.6)	
15–24	10 (9.7)	474 (24.1)	
25–34	27 (26.2)	510 (25.9)	
35–44	33 (32.0)	388 (19.7)	
45–54	27 (26.2)	304 (15.5)	
55–64	4 (3.9)	131 (6.7)	
65+	1 (1.0)	68 (3.5)	
Respiratory Symptoms *n*(%)			
Cough	23 (22.3)	404 (20.6)	0.665
Sputum	7 (6.8)	140 (7.1)	0.900
Wheezing	11 (10.7)	131 (6.7)	0.116
Shortness of breath	18 (17.5)	177 (9.0)	0.004
Risk factors and co-morbid conditions *n* (%)			
History of smoking	13 (12.6)	155 (7.9)	0.086
Exposure to bio-mass	24 (23.3)	385 (19.6)	0.357
Ever been treated for TB	10 (9.7)	30 (1.5)	< 0.001
Allergy	42 (40.8)	721 (36.7)	0.400
Family history of asthma	15 (14.6)	220 (11.2)	0.295

*PLHIV* people living with HIV*, TB* tuberculosis

### Prevalence of asthma by HIV status

**Fig. 1:**
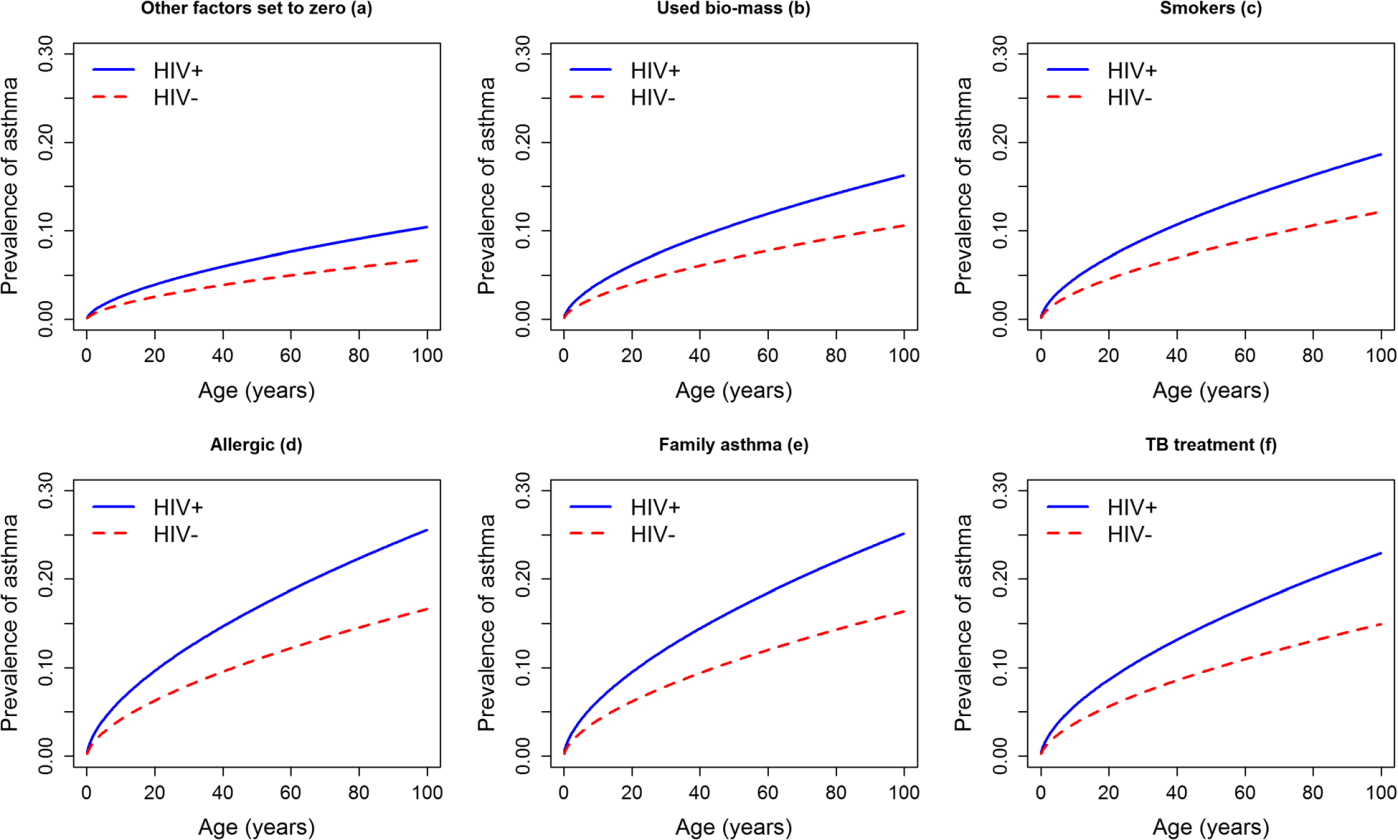
Prevalence of asthma by age stratified by HIV status and other asthma risk factors: **a** Prevalence of asthma by age and HIV status keeping all other factors at zero. **b** Prevalence of asthma by age and HIV status among participants exposed to biomass smoke (**c**) Prevalence of asthma by age and HIV status among smokers. **d** Prevalence of asthma by age and HIV status among participants with history of allergy. **e** Prevalence of asthma by age and HIV status among participants with family history of asthma **f**) Prevalence of asthma by age and HIV status among participants with history of TB treatment

**Fig. 2:**
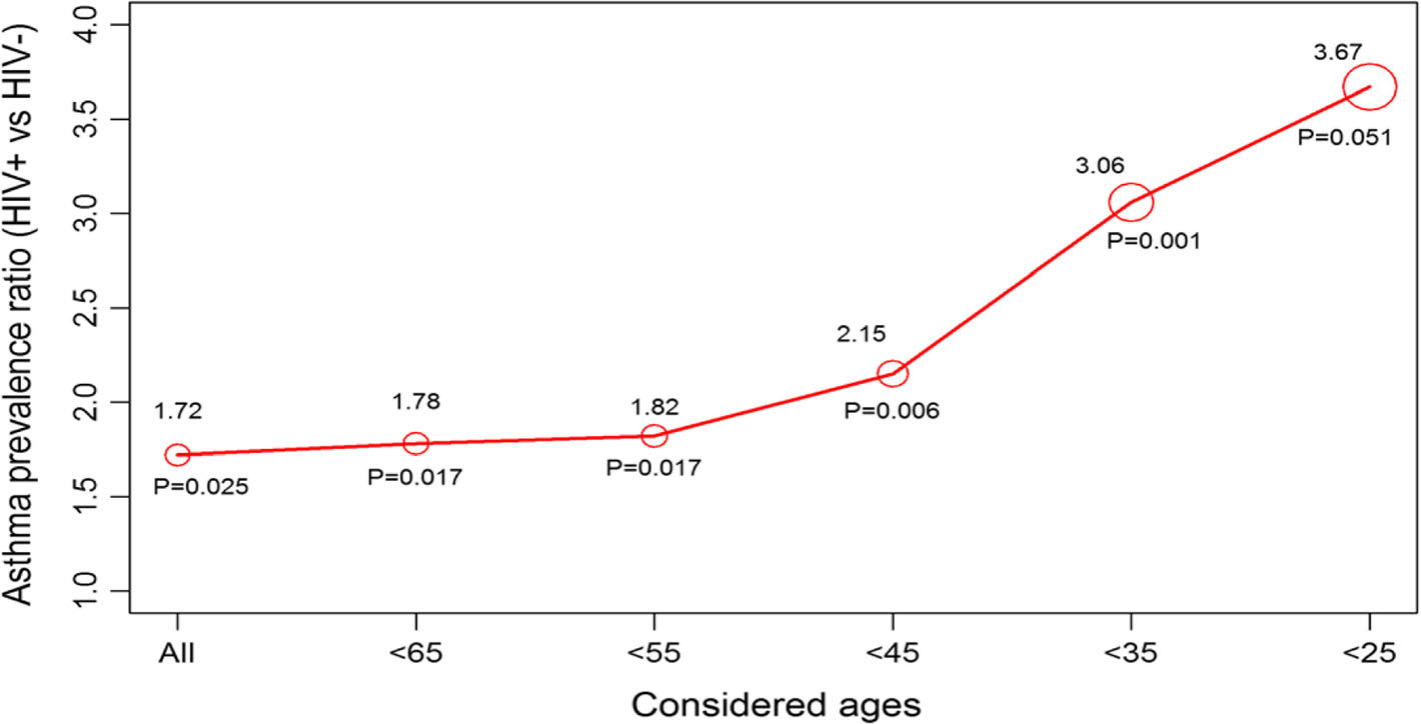
Asthma prevalence ratios (HIV+ vs HIV-) considering different ages of participants

**Fig. 3:**
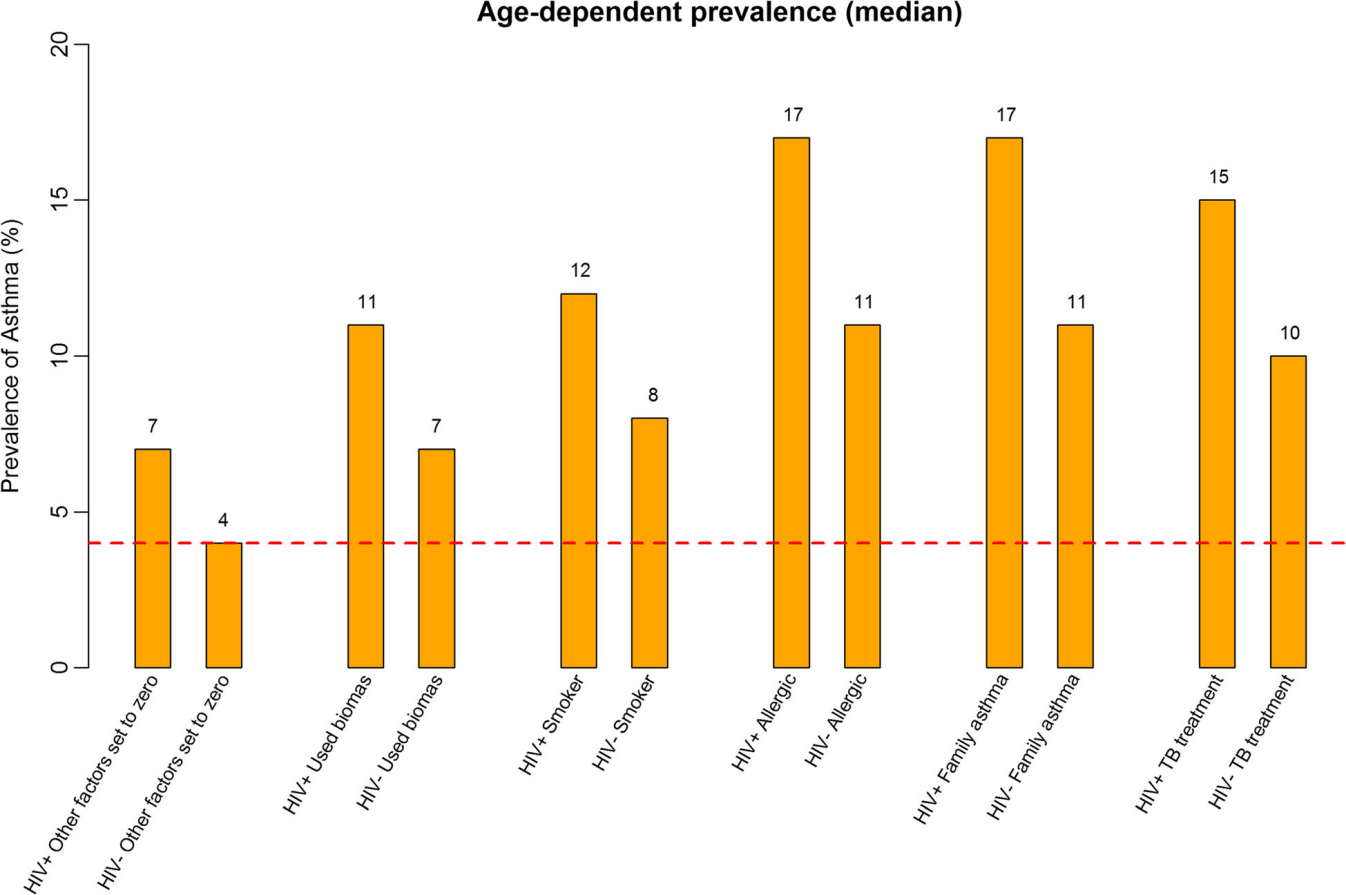
Asthma prevalence considering different known asthma risk factors (all *p*-values < 0.001)

The prevalence of asthma among PLHIV was 15.5% while that among those without HIV was 9.1%, PR 1.72 (95% CI: 1.07–2.75, *p* = 0.025). After adjusting for gender, age, tobacco smoking, biomass exposure, TB treatment, family history of asthma and allergy, the PR ratio decreased to 1.54 (95% CI: 0.94–2.51, *p* = 0.085), Table 2. A high prevalence of asthma among PLHIV was maintained at all ages irrespective of absence or presence of other risk factors of tobacco smoking, biomass use, allergy, family history of asthma and previous TB treatment (Fig. 1). Considering only participants younger than 35 years the prevalence of asthma was still higher among PLHIV, PR 3.06 (Fig. 2). The prevalence of asthma among participants with known HIV status and unknown HIV status did not differ significantly (9.4% vs.9.0%, *p* = 0.689), Table 3. HIV modified the association between asthma and other asthma risk factors, positive vs. negative: tobacco smoking (12% vs.8%, *p* = < 0.001) biomass use (11% vs. 7%, *p* = < 0.001), allergy (17% vs. 11%, *p* = < 0.001), family history asthma (17% vs. 11%, *p* = < 0.001) and TB treatment (15% vs. 10%, *p* = < 0.001), Fig. 3.

**Table 2:** Asthma prevalence by HIV and potential confounding factors

	*n* (%)	Prevalence of asthma %	Crude PR (95% CI)	*p*	Adjusted PR^b^ (95% CI)	*p*-value
Characteristic						
HIV status						
Infected	103 (5.0)	15.5	1.72 (1.07–2.75)	0.025	1.54 (0.94–2.51)	0.085
Uninfected	1966 (95.0)	9.1	Reference		Reference	
Gender						
Female	1275 (61.6)	9.9	1.15 (0.87–1.53)	0.319	1.24 (0.92–1.68)	0.159
Male	794 (38.4)	8.6	Reference		Reference	
Smoking						
Yes	168 (8.1)	16.7	1.91 (1.32–2.76)	0.001	1.79 (1.23–2.60)	0.002
No	1901 (91.9)	8.7	Reference		Reference	
Biomass use						
Yes	409 (19.8)	14.2	1.74 (1.31–2.32)	< 0.001	1.56 (1.18–2.07)	0.002
No	1659 (80.2)	8.1	Reference		Reference	
TB treatment						
Yes	40 (1.9)	22.5	2.46 (1.36–4.45)	0.003	2.20 (1.26–3.84)	0.005
No	2026 (98.1)	9.1	Reference		Reference	
Family history of asthma						
Yes	235 (11.4)	23.8	3.16 (2.39–4.18)	< 0.001	2.41 (1.81–3.23)	< 0.001
No	1832 (88.6)	7.5	Reference		Reference	
Allergy						
Yes	763 (36.9)	16.0	2.90 (2.20–3.83)	< 0.001	2.45 (1.85–3.26)	< 0.001
No	1306 (63.1)	5.5	Reference		Reference	
TB/HIV (HIV infected and history of TB treatment)						
Yes	10 (0.5)	10.0	1.07 (0.17–6.89)	0.946	0.20 (0.02–1.66)	0.135
No	2059 (99.5)	9.4	Reference		Reference	
Age in years^a^			2.09 (1.52–2.88)	< 0.001	1.83 (1.30–2.58)	0.001

^a^log transformed due to skewness, ^b^Adjusted for age, gender, smoking, biomass smoke exposure, allergy, history of TB and family history of asthma, *CI* confidence interval, *TB* tuberculosis, *PR* prevalence ratio

**Table 3:** Participants characteristics by HIV status (known vs. unknown status)

	HIV status known (*N* = 2069)	HIV status unknown (*N* = 1347)	*P*-value
Characteristic			
Male gender	794 (38.4)	533 (39.6)	0.484
Age groups			< 0.001
< 15	92 (4.5)	280 (20.8)	
15–24	484 (23.4)	400 (29.7)	
25–34	537 (26.0)	144 (10.7)	
35–44	421 (20.4)	156 (11.6)	
45–54	331 (16.0)	144 (10.7)	
55–64	135 (6.5)	90 (6.7)	
65+	69 (3.3)	133 (9.9)	
Respiratory Symptoms			
Cough	427 (20.7)	284 (21.1)	0.751
Sputum	147 (7.1)	110 (8.2)	0.250
Wheezing	142 (6.9)	84 (6.2)	0.471
Shortness of breath	195 (9.4)	114 (8.5)	0.337
Risk factors and co-morbid conditions			
History of smoking	168 (8.1)	74 (5.5)	0.004
Exposure to bio-mass	409 (19.8)	289 (21.5)	0.235
Ever been treated for TB	40 (1.9)	10 (0.7)	0.005
Allergy	763 (36.9)	433 (32.2)	0.005
Family history of asthma	235 (11.4)	142 (10.6)	0.455
Asthma			
Positive	194 (9.4)	121 (9.0)	0.698

*TB* tuberculosis

### Prevalence of asthma by other factors

Other factors associated with increased prevalence of asthma in this study were biomass use adjusted PR 1.56 (95% CI: 1.18–2.07, *p* = 0.002), tobacco smoking 1.79 (95% CI: 1.23–2.60, *p* = 0.002), history of TB treatment 2.20 (95% CI: 1.26–3.84, *p* = 0.005), family history of asthma 2.41 (95% CI: 1.81–3.23, *p* = < 0.001), and allergy 2.45 (95% CI: 1.85–3.26, *p* = < 0.001), Table 2.

## Discussion

This study has established that the prevalence of asthma among PLHIV is higher than among those without HIV and that HIV modifies the associations of asthma with tobacco smoking, biomass use, TB, allergy and family history of asthma. The study also shows that the only respiratory symptom more prevalent in PLHIV than without PLHIV is breathlessness. In terms of asthma risk factors the study found that PLHIV have high rates of tobacco smoking and history of TB treatment.

A higher prevalence of asthma among PLHIV has been reported in previous studies in clinic-based studies in high income with low HIV prevalence [6–12]. In 248 HIV infected and 236 HIV uninfected males, the prevalence of wheezing was 54.4%, vs. 21.2%, *p* < 0.001 [9] while in a study comparing 14,005 HIV infected patients with age matched HIV uninfected controls in Canada, the PR for asthma was 1.31 (95% CI 1.20–1.43) [12]. In another study among 223 HIV patients in the USA, the prevalence of doctor diagnosed asthma was 20.6 compared to 8.2% in the general population [13]. Although after adjusting for other risk factors of asthma such as smoking, biomass smoke, TB, allergy the PR ratio for asthma in PLHIV reduced to 1.54 with a trend *p*-value of 0.085. The results are however line with the findings from the studies above which increases the possibility that HIV is associated with asthma even in the present study.

We investigated the effect HIV had on the risk of asthma from other asthma risk factors. We found that PLHIV had higher rates of smoking as previously reported [25, 26]. The prevalence of asthma among tobacco smoking PLHIV was 12.6% compared to 7.9% of smokers who were not living with HIV. The finding of high smoking rates among PLHIV in this study has been reported in previous studies [25, 26]. This finding therefore calls for heightened efforts to reduce smoking among PLHIV.”

We compared respiratory symptoms between HIV infected and HIV uninfected participants and found that there were no differences except for breathlessness. George et al. found that HIV infected persons had high rates of most respiratory symptoms [21]. We cannot explain why we failed to observe these differences in respiratory symptoms apart from breathlessness. The excess breathlessness in the HIV infected participants might be due to interstitial and diffusion derangements that are so prevalent among HIV infected persons [27–29]. Gingo et al. in a cross-sectional analysis of 158 HIV-infected individuals without acute respiratory symptoms or infection found that 55 (34.8%) participants had a significantly reduced DL_CO_ (< 60% predicted) [29].

This study had limitations. Most notably the use of self-report to determine HIV infection. Self-report as a means of determining HIV status has been reported to have low sensitivity but high specificity [30, 31]. Among older adults (≥40 years) in South Africa, Rohr et al. report a sensitivity of HIV self-report of 51.2% (95% CI: 48.2–54.3) and specificity was 99.0% (95% CI: 98.7–99.4) [30, 31]. The low sensitivity of self-report may also be present in our study, the self-reported prevalence of HIV in our survey is 3.1%, which is much lower than the national prevalence of 6.2% [4]. We believe that differential classification of HIV by asthma status is unlikely, but if present would likely tend to underestimate the prevalence ratio if patients without HIV are less likely to report HIV status. We performed further analysis on our data comparing demographic characteristics and asthma prevalence between participants with known HIV status and those with unknown HIV status, Table 3. As can be seen in this table the prevalence of asthma among these two groups did not significantly differ.

Another limitation of our study is that asthma was diagnosed based on history or current wheeze, prior physician diagnosis and being currently on asthma medications. However the methods used in this study are the standard methods that are used in most asthma surveys including the ISAAC), ECRHS)and the WHO health survey [1, 22, 23]. We recognise that wheezing could be due to other diseases such as chronic obstructive pulmonary disease (COPD). Therefore, it is possible that some of the participants classified as asthma could have had COPD which is also known to be associated with HIV. We performed sensitivity analysis considering only participants who are younger than 35 years, the age below which the diagnosis of COPD is unlikely and found that the prevalence of asthma in age group was still higher among the HIV infected participants (Fig. 1 left panel) implying that the association between asthma and HIV in this study might be a true one. There are however other conditions that can cause wheezing such as bronchiectasis, heart failure and mechanical airway obstruction [32, 33] that we could not exclude although these conditions are deemed to be rare.

Despite the limitations, our findings have several scientific, healthcare and programmatic implications. Firstly, HIV care programs need to build capacity for diagnosis of asthma and its management. At the same time there may be need to test for HIV among asthma patients in high HIV burden settings and to know their ART status and the medications they are taking. Asthma and HIV co-morbidity can be associated with complexities that can arise while managing the two diseases notably drug-drug interactions and increased rates of adverse events as can occur with corticosteroids used in the management of asthma. Corticosteroids use in HIV infected persons has been associated with adverse outcomes such as the development of cancers like Kaposi’s sarcoma and TB [34–36]. Another key drug-drug interaction to consider is that between protease inhibitors and corticosteroids, which is mediated through the effects of PIs on the cytochrome CYP450 3A4 drug metabolism pathway [37, 38]. This interaction can lead to complications such as Cushing’s syndrome, hypertension and poor CD4 cell count recovery [38]. We found that HIV modified the risk of asthma from other risk factors. This calls for vigorous prevention of these risk factors among PLHIV. PLHIV in this study had higher rates of tobacco smoking. This calls for heightened efforts to reduce smoking among PLHIV since they may be more likely to develop tobacco associated lung diseases. In terms of science, our findings call for more research on the impact of HIV on asthma in high HIV burden settings and the mechanistic pathways of the HIV asthma interaction especially in settings with high rates of factors like TB and biomass smoke exposure. The impact of HIV on asthma prognosis also needs to be studied.

## Conclusion

In conclusion, the prevalence of asthma among PLHIV in this survey is higher than among those without HIV. HIV also modifies the risk of asthma from other asthma risk factors such as tobacco smoking, TB, exposure to biomass, allergy and family history of asthma. PLHIV should be assessed for asthma and asthma patients should undergo HIV testing.

## Abbreviations

ART: Antiretroviral therapy
CD4: Cluster of differentiation 4
CI: Confidence interval
COPD: Chronic obstructive pulmonary disease
CY: Cytochrome
ECRHS: European community respiratory health survey
HIV: Human immune deficiency virus
HHs: Households
IgE: Immunoglobulin E
IL-4: Interleukin-4
ISAAC: International study of asthma and allergies in children
LMICs: Low and middle-income countries
NCDs: Non-communicable diseases
PLHIV: People living with HIV
PR: Prevalence ratio
TB: Tuberculosis
UNAS: Uganda National asthma survey
USA: Unites States of America
WHO: World Health Organization

## References

[1] Stanojevic S, Moores G, Gershon AS, Bateman ED, Cruz AA, To T (2012). Global asthma prevalence in adults: findings from the cross-sectional world health survey. BMC Public Health.

[2] Marais BJ (2011). Childhood tuberculosis: epidemiology and natural history of disease. The Indian Journal of Pediatrics.

[3] Adeloye D, Chan KY, Rudan I, Campbell H (2013). An estimate of asthma prevalence in Africa: a systematic analysis. Croatian medical journal.

[4] Ayles H, Muyoyeta M, Du Toit E, Schaap A, Floyd S, Simwinga M (2013). Effect of household and community interventions on the burden of tuberculosis in southern Africa: the ZAMSTAR community-randomised trial. Lancet.

[5] Morgan BW, Siddharthan T, Grigsby MR, Pollard SL, Kalyesubula R, Wise RA (2018). Asthma and allergic disorders in Uganda: a population-based study across urban and rural settings.

[6] Puri A, Gingo M, Morris A. Asthma in HIV-infected population: a review of respiratory symptoms, pulmonary function abnormalities and pathophysiology. Epidemiol. 2014;4:164. 10.4172/2161-1165.1000164.

[7] Wallace JM, Stone GS, Browdy BL, Tashkin DP, Hopewell PC, Rosen MJ, et al. Nonspecific airway hyperresponsiveness in HIV disease. Chest 1997;111(1):121–127. 10.1378/chest.111.1.121 8996005

[8] O'Donnell CR, Bader MB, Zibrak JD, Jensen WA, Rose RM (1988). Abnormal airway function in individuals with the acquired immunodeficiency syndrome. Chest.

[9] Poirier CD, Inhaber N, Lalonde RG, Ernst P (2001). Prevalence of bronchial hyperresponsiveness among HIV-infected men. Am J Respir Crit Care Med.

[10] Crothers K, Huang L, Goulet JL, Goetz MB, Brown ST, Rodriguez-Barradas MC (2011). HIV infection and risk for incident pulmonary diseases in the combination antiretroviral therapy era. Am J Respir Crit Care Med.

[11] Lin RY, Lazarus TS (1995). Asthma and related atopic disorders in outpatients attending an urban HIV clinic. Ann Allergy Asthma Immunol.

[12] Kendall CE, Wong J, Taljaard M, Glazier RH, Hogg W, Younger J (2014). A cross-sectional, population-based study measuring comorbidity among people living with HIV in Ontario. BMC Public Health.

[13] Gingo MR, Wenzel SE, Steele C, Kessinger CJ, Lucht L, Lawther T, et al. Asthma diagnosis and airway bronchodilator response in HIV-infected patients. Journal of allergy and clinical immunology. 2012;129(3):708–14. e8. 10.1016/j.jaci.2011.11.015 PMC3294124 22177327

[14] Drummond MB, Kirk GD, Astemborski J, Marshall MM, Mehta SH, McDyer JF, et al. Association between obstructive lung disease and markers of HIV infection in a high-risk cohort. Thorax. 2011:thoraxjnl-2011-200702. 10.1136/thoraxjnl-2011-200702 PMC4135473 22090038

[15] Wright DN, Nelson RP, Ledford DK, Fernandez-Caldas E, Trudeau WL, Lockey RF (1990). Serum IgE and human immunodeficiency virus (HIV) infection. J Allergy Clin Immunol.

[16] Thuesen B, Husemoen L, Hersoug LG, Pisinger C, Linneberg A (2009). Insulin resistance as a predictor of incident asthma-like symptoms in adults. Clin Exp Allergy.

[17] Drummond MB, Merlo CA, Astemborski J, Marshall MM, Kisalu A, Mcdyer JF (2013). The effect of HIV infection on longitudinal lung function decline among injection drug users: a prospective cohort. AIDS (London, England).

[18] Gingo MR, Morris A (2013). Pathogenesis of HIV and the lung. Current HIV/AIDS Reports.

[19] Kidd P (2003). Th1/Th2 balance: the hypothesis, its limitations, and implications for health and disease. Altern Med Rev.

[20] Deo SS, Mistry KJ, Kakade AM, Niphadkar PV (2010). Role played by Th2 type cytokines in IgE mediated allergy and asthma. Lung India.

[21] George MP, Kannass M, Huang L, Sciurba FC, Morris A (2009). Respiratory symptoms and airway obstruction in HIV-infected subjects in the HAART era. PLoS One.

[22] Asher M, Anderson H, Stewart A, Crane J, Ait-Khaled N, Anabwani G (1998). Worldwide variations in the prevalence of asthma symptoms: the international study of asthma and allergies in childhood (ISAAC). Eur Respir J.

[23] Fitzpatrick C, Floyd K (2012). A systematic review of the cost and cost effectiveness of treatment for multidrug-resistant tuberculosis. PharmacoEconomics.

[24] Coutinho L, Scazufca M, Menezes PR (2008). Methods for estimating prevalence ratios in cross-sectional studies. Revista de saude publica.

[25] Mdege ND, Shah S, Ayo-Yusuf OA, Hakim J, Siddiqi K (2017). Tobacco use among people living with HIV: analysis of data from demographic and health surveys from 28 low-income and middle-income countries. Lancet Glob Health.

[26] Mdodo R, Frazier EL, Dube SR, Mattson CL, Sutton MY, Brooks JT (2015). Cigarette smoking prevalence among adults with HIV compared with the general adult population in the United States: cross-sectional surveys. Ann Intern Med.

[27] Doffman SR, Miller RF (2013). Interstitial lung disease in HIV. Clin Chest Med.

[28] Semenzato G, Agostini C (1995). HIV-related interstitial lung disease. Curr Opin Pulm Med.

[29] Gingo MR, He J, Wittman C, Fuhrman C, Leader JK, Kessinger C (2014). Contributors to diffusion impairment in HIV-infected persons. Eur Respir J.

[30] Rohr JK, Xavier Gómez-Olivé F, Rosenberg M, Manne-Goehler J, Geldsetzer P, Wagner RG, et al. Performance of self-reported HIV status in determining true HIV status among older adults in rural South Africa: a validation study. Journal of the International AIDS Society. 2017;20(1). 10.7448/IAS.20.1.21691 PMC5577734 28782333

[31] Validity of data on self-reported hiv status and implications for measurement of arv coverage in malawi. [cited 2018 June 7]; Available from: https://dhsprogram.com/pubs/pdf/WP81/WP81.pdf.

[32] Clerf L (1953). Differential diagnosis of wheezing respiration. J Am Geriatr Soc.

[33] Lillington GA, Lin H-w (1994). Differential diagnosis of asthma in adults asthma, occult asthma, and Pseudoasthma.

[34] Lee C-H, Kim K, Hyun MK, Jang EJ, Lee NR, Yim J-J (2013). Use of inhaled corticosteroids and the risk of tuberculosis. Thorax.

[35] Brassard P, Suissa S, Kezouh A, Ernst P (2011). Inhaled corticosteroids and risk of tuberculosis in patients with respiratory diseases. Am J Respir Crit Care Med.

[36] Gill PS, Loureiro C, Bernstein-Singer M, Rarick MU, Sattler F, Levine AM (1989). Clinical effect of glucocorticoids on Kaposi sarcoma related to the acquired immunodeficiency syndrome (AIDS). Ann Intern Med.

[37] Foisy M, Yakiwchuk E, Chiu I, Singh A (2008). Adrenal suppression and Cushing's syndrome secondary to an interaction between ritonavir and fluticasone: a review of the literature. HIV medicine..

[38] Saberi P, Phengrasamy T, Nguyen DP (2013). Inhaled corticosteroid use in HIV-positive individuals taking protease inhibitors: a review of pharmacokinetics, case reports and clinical management. HIV medicine.

